# Supplementary material for: Effect of Protein Conformation and AMP Protonation State on Fireflies’ Bioluminescent Emission
Source: Molecules. 2019 Apr 20;24(8):1565. doi: 10.3390/molecules24081565 (PMC6514813; doi:10.3390/molecules24081565)
Supplement: Supplementary file 1 [file molecules-24-01565-s001.pdf]

Supplementary Materials

Effect of protein conformation and AMP protonation state on fireflies' bioluminescent emission

Cristina García-Iriepa[1,] and Isabelle Navizet[1,*]*

[1]Université Paris-Est, Laboratoire Modélisation et Simulation Multi Échelle, MSME UMR 8208 CNRS, UPEM, 5 bd Descartes, 77454 Marne-la-Vallée, France

Content

1. Statistical representation of waters around oxyluciferin.
2. Molecular orbitals involved in the vertical transition.
3. Protein active site comparison from the X-Ray structures.
4. Histograms oscillator strengths.
5. DFT functionals benchmark.
6. Protonated histidine residues.
7. Cartesian coordinates QM region.

1. Statistical representation of waters around oxyluciferin.

To check if less water molecules access the protein active site when considering the closed protein conformation (4G37), compared to the open one (4G36), the number of water molecules around oxyluciferin during the MD simulation has been calculated. In particular, from the mdcrd file of Amber, the watershell option of cpptraj tool has been used for this aim. Regarding phenolate-keto, we have selected the oxygen atoms of the phenolate and keto moieties (O1 and O2, respectively in Figures 2 and 3), and the phosphorus atom of the AMP phosphate group atom. This way we cover all the oxyluciferin structure and so we can analyze if the same behavior is found in all the protein active site or only in certain sides of it.

By analyzing the histograms in Figure S1, we can conclude that fewer water molecules are placed around oxyluciferin in the closed protein conformation than in the open one. It is the case near the phenolate moiety (Figure S1a), close the keto part (Figure S1b) and around AMPH (Figure S1c).

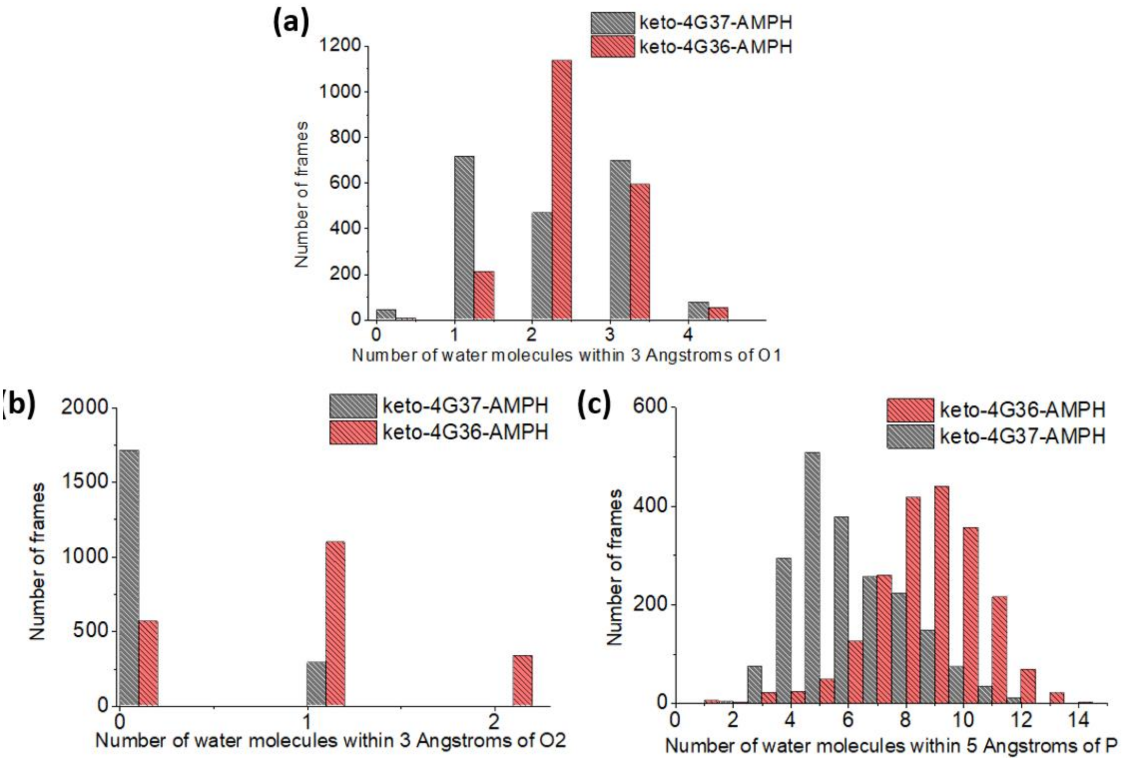

Figure S1. Histograms representing the number of water molecules during the MD simulation within (a) 3 Ångstroms of the phenolate oxygen atom O1, (b) 3 Ångstroms of the keto oxygen atom (O2) and (c) 5 Ångstroms of the phosphorus atom of the AMP phosphate group.

2. Molecular orbitals involved in the vertical transition.

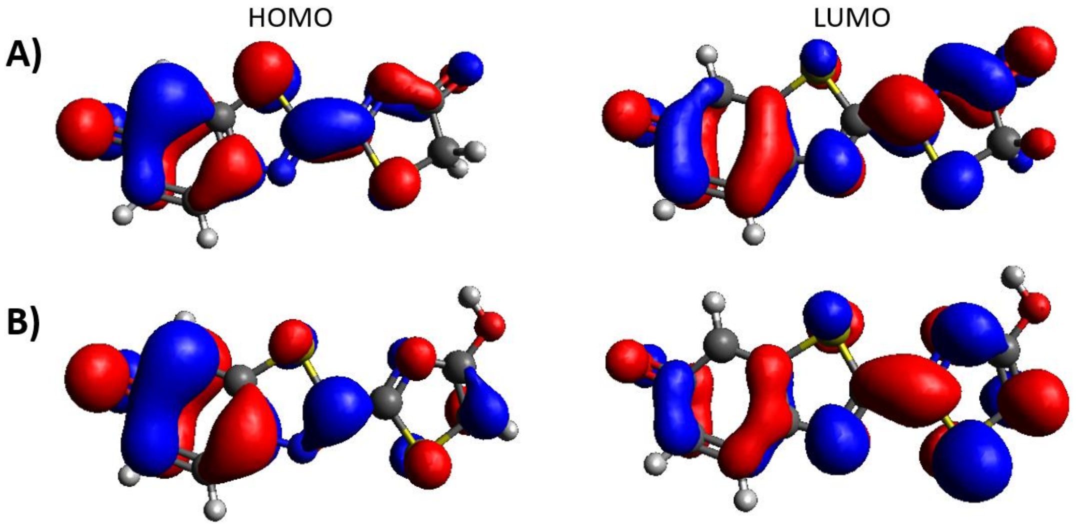

Figure S2. HOMO and LUMO orbitals involved in the emission transition for one selected snapshot of A) keto-4G36-AMPH and B) enol-4G36-AMPH.

3. Protein active site comparison from the X-Ray structures.

The active sites of 4G37 (brown sticks in Figure S3) and 4G36 pdb structures have been compared in order to analyze the possible differences on some amino acid positions. In Figure S3 we observe that in the surrounding of the phenolate moiety, both structures almost overlap as they are quite similar (beige shadow). Whereas, in the AMP side we find significative differences (blue shadow). For instance, for 4G37 (brown sticks) three amino acids (GLN448, LEU441 and LYS439) are close to AMP, whereas for the open conformation 4G36, water molecules are placed in this position. Moreover, we see that ARG437 is further from AMP for the 4G37 (brown sticks) compared to 4G36, explaining the fact that when AMP is deprotonated (considering the phenolate-keto oxyluciferin), no interaction is observed for the closed conformation (4G37) but a strong one was found for the open one (4G36).

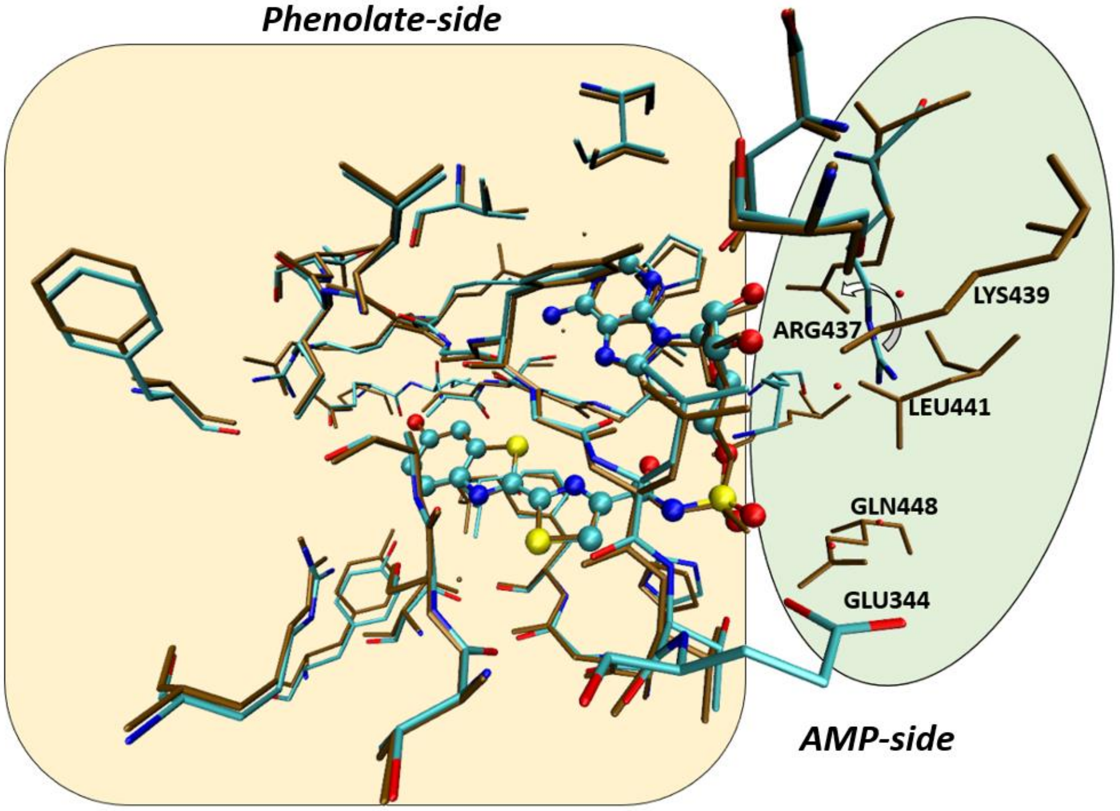

Figure S3. Graphical comparison of the active sites of the open (4G36, in atom color code C, cyan; O, red; N, blue; S, yellow) and closed (4G37, in brown) protein conformations. The active site is divided into the phenolate and AMP sides. Remarkable amino acids are given with the three letters code and their corresponding number.

4. Histograms oscillator strengths.

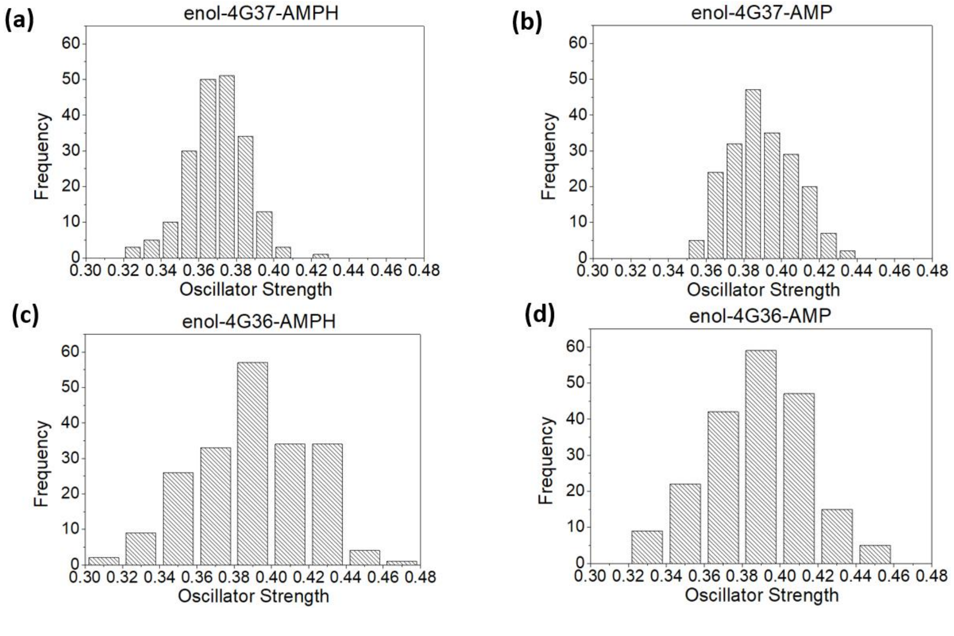

Figure S4. Histogram of the oscillator strength computed of the emission transition at the QM/MM level for 200 snapshots of the phenolate-enol form.

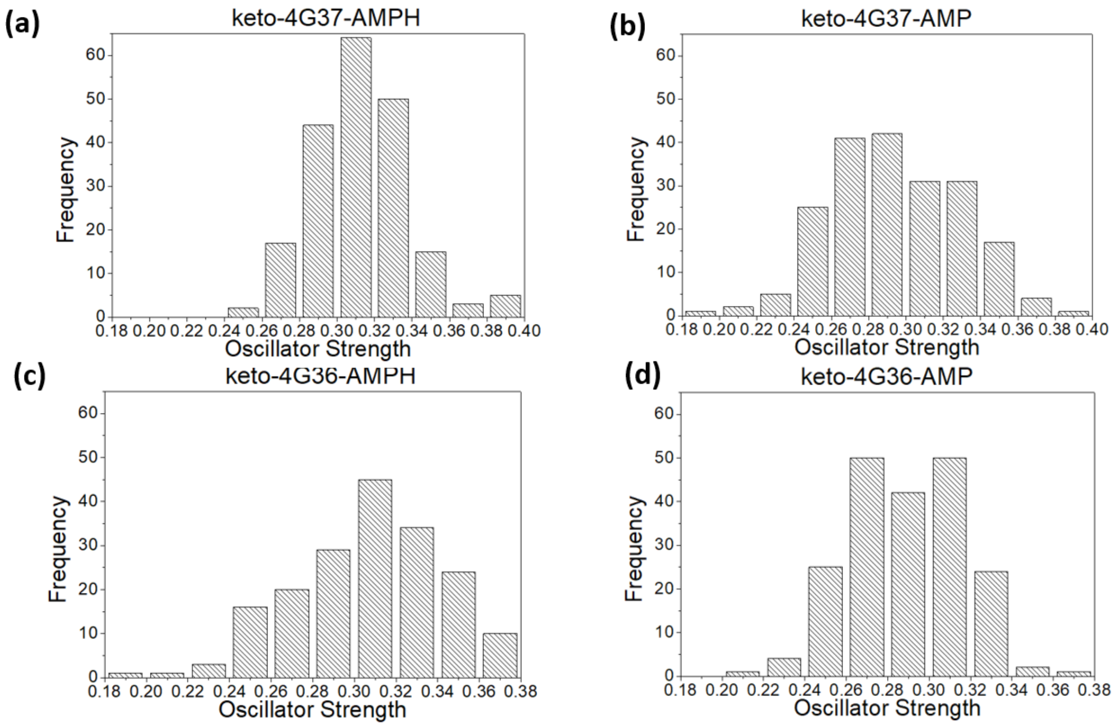

Figure S5. Histogram of the oscillator strength computed of the emission transition at the QM/MM level for 200 snapshots of the phenolate-keto form.

5. DFT functionals benchmark.

Table S1. Emission energy computed at the QM/MM level after optimization in the first excited state of one snapshot located in the maximum of the emission spectra with the B3LYP, CAM_B3LYP and M062X functionals.

	Enol			Keto		
	B3LYP	CAM-B3LYP	M062X	B3LYP	CAM-B3LYP	M062X
4G36-AMPH	2.296 (540)	2.551 (486)	2.566 (482)	2.127 (583)	2.520 (492)	2.495 (497)
4G36-AMP	2.313 (536)	2.583 (480)	2.596 (478)	2.123 (584)	2.465 (503)	2.460 (504)
4G37-AMPH	2.344 (529)	2.638 (470)	2.661 (466)	2.179 (569)	2.532 (490)	2.540 (488)
4G37-AMP	2.431 (510)	2.707 (458)	2.737 (453)	2.313 (536)	2.737 (453)	2.755 (450)

6. Protonated histidine residues.

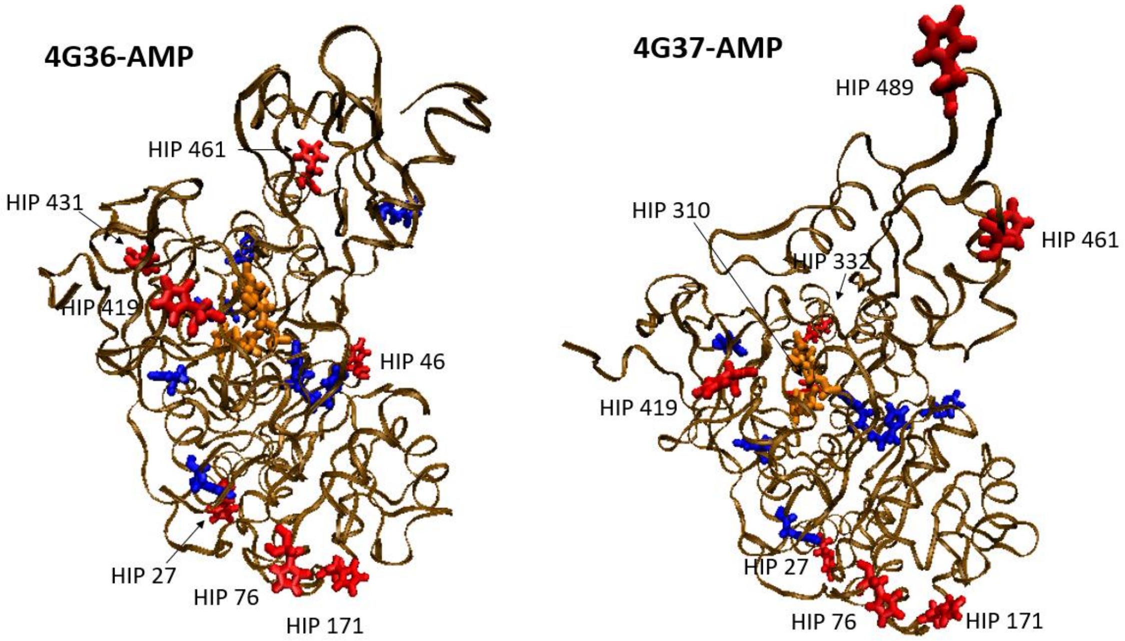

Figure S6. Protonated histidine residues depicted in red for both the open (4G36) and closed (4G37) protein conformations (see Material and Methods section in the main text for further details). The non-protonated histidine residues are depicted in blue. Oxyluciferin and AMPH are depicted in orange.

7. Cartesian coordinates QM region.

The cartesian coordinates of one representative snapshot (around the emission maxima) for the systems under study. Only the coordinates of the QM region are given.

Keto-4G36-AMPH	Keto-4G36-AMP
N 0.125980 -1.313939 0.597147	N -0.069106 -1.228714 0.599201
C 0.023936 -0.110962 0.004872	C -0.017785 -0.047086 -0.059966
O -0.950948 0.657872 0.068999	O -0.928292 0.782159 -0.159967
C 1.256473 0.282820 -0.842679	C 1.351405 0.272614 -0.685266
H 0.980929 0.462299 -1.882332	H 1.303124 0.371767 -1.770721
H 1.682719 1.204522 -0.448594	H 1.669660 1.226025 -0.272182
S 2.423886 -1.104755 -0.733523	S 2.460739 -1.090789 -0.236129
C 1.247466 -1.961877 0.304497	C 1.081866 -1.866822 0.594752
C 1.556336 -3.268788 0.729976	C 1.344640 -3.142537 1.165467
N 2.579630 -4.015930 0.356835	N 2.404845 -3.860921 0.885831
C 2.570921 -5.236215 0.994650	C 2.402931 -5.046371 1.589069
C 3.504896 -6.283701 0.882319	C 3.379593 -6.053402 1.537694
H 4.340530 -6.158121 0.200448	H 4.241754 -5.901467 0.895468
C 3.388558 -7.448298 1.622443	C 3.257802 -7.205657 2.282513
H 4.115595 -8.243032 1.511167	H 4.009879 -7.981866 2.263669

C 2.300012 -7.678602 2.544633	C 2.117865 -7.454919 3.104995
O 2.120993 -8.714573 3.219575	O 1.958995 -8.535728 3.732978
C 1.330423 -6.582802 2.656159	C 1.124210 -6.394097 3.204768
H 0.496004 -6.708049 3.333509	H 0.257748 -6.558790 3.835186
C 1.487920 -5.424757 1.892956	C 1.298385 -5.223500 2.447155
S 0.463740 -4.039112 1.907941	S 0.248744 -3.848899 2.362484
**Keto-4G37-AMPH**	**Keto-4G37-AMP**
N 1.218531 -0.474935 -0.546171	N 1.355983 -0.128767 -0.272469
C 0.019095 -0.055045 -0.047981	C 0.040901 0.123094 0.006412
O -0.593608 0.952449 -0.388700	O -0.817607 0.452750 -0.804545
C -0.549805 -0.962560 1.061383	C -0.313132 -0.066685 1.499604
H -0.816385 -0.361403 1.928565	H -0.758207 0.844982 1.895520
H -1.450897 -1.456335 0.701233	H -1.019319 -0.888720 1.613626
S 0.742393 -2.180212 1.443795	S 1.240771 -0.476401 2.362401
C 1.713604 -1.503124 0.097473	C 2.055440 -0.466718 0.784910
C 2.979470 -2.127498 -0.147225	C 3.426017 -0.891835 0.717826
N 3.458421 -3.136040 0.548034	N 4.163819 -1.292682 1.722505
C 4.656664 -3.589807 0.007289	C 5.410494 -1.720532 1.259002
C 5.397698 -4.697630 0.438633	C 6.465729 -2.226182 2.032599
H 4.993197 -5.303169 1.240761	H 6.346225 -2.269022 3.108579
C 6.593549 -5.035590 -0.159302	C 7.646440 -2.652974 1.450978
H 7.141256 -5.908860 0.167429	H 8.480702 -3.012818 2.041298
C 7.116694 -4.321711 -1.282194	C 7.831360 -2.599229 0.044354
O 8.202477 -4.625823 -1.861237	O 8.914843 -2.951350 -0.535753
C 6.321985 -3.198164 -1.756219	C 6.755475 -2.063786 -0.754463
H 6.667295 -2.662231 -2.631442	H 6.892054 -2.001665 -1.826956
C 5.120199 -2.861770 -1.093044	C 5.581185 -1.624914 -0.123345
S 4.004169 -1.587544 -1.476080	S 4.181829 -0.940547 -0.876084
**Enol-4G36-AMPH**	**Enol-4G36-AMP**
N 0.223439 -0.886871 0.994882	N -0.260482 -0.770608 0.929759
C 0.065784 0.085681 0.084043	C 0.110448 0.048925 -0.066762
O -0.767426 1.137464 0.366765	O -0.359125 1.340504 -0.061250
H -0.884649 1.129738 1.329834	H -0.142294 1.706342 -0.966317
C 0.735754 -0.042181 -1.109162	C 0.945647 -0.457358 -1.039837
H 0.815479 0.653229 -1.924854	H 1.451466 0.077390 -1.826287
S 1.621733 -1.529842 -1.095467	S 1.269873 -2.128192 -0.706719
C 1.043285 -1.833233 0.536687	C 0.288835 -1.974947 0.749297
C 1.471669 -3.004261 1.251494	C 0.223125 -3.173524 1.539145
N 2.414390 -3.830523 0.859705	N 0.828851 -4.273126 1.148368
C 2.549063 -4.901371 1.766059	C 0.791448 -5.241227 2.157483
C 3.494575 -5.944203 1.691131	C 1.438508 -6.490580 2.133722
H 4.239906 -5.934779 0.903947	H 2.003594 -6.760285 1.246686
C 3.475118 -6.970317 2.600968	C 1.360927 -7.345545 3.210893
H 4.196274 -7.775688 2.555325	H 1.838954 -8.316587 3.186155
C 2.473645 -7.058767 3.633981	C 0.605984 -7.021932 4.391276
O 2.389177 -8.058214 4.396282	O 0.469279 -7.824343 5.373816
C 1.558431 -5.943939 3.747571	C -0.036945 -5.726641 4.412354
H 0.792357 -5.987458 4.510121	H -0.599545 -5.454447 5.296751
C 1.640340 -4.898719 2.825224	C 0.074175 -4.885564 3.301971
S 0.669654 -3.437746 2.768465	S -0.581725 -3.264253 3.104498
**Enol-4G37-AMPH**	**Enol-4G37-AMP**
N 1.277293 0.089136 -0.501840	N 1.225301 -0.200200 -0.602289
C 0.031915 -0.020005 -0.017832	C 0.038212 -0.072489 0.033061
O -0.943698 0.747656 -0.580688	O -0.844180 0.847752 -0.413263
H -1.764754 0.579186 -0.070917	H -1.621090 0.960834 0.206110

C	-0.164879	-0.900327	1.026638
H	-1.091151	-1.174299	1.496229
S	1.342071	-1.624646	1.480455
C	2.099575	-0.714577	0.171243
C	3.494328	-0.916804	-0.039812
N	4.257147	-1.573258	0.818404
C	5.539480	-1.749351	0.280955
C	6.570787	-2.480447	0.889422
H	6.371835	-2.997798	1.820552
C	7.808254	-2.540634	0.302999
H	8.623673	-3.077042	0.765484
C	8.100652	-1.885456	-0.935582
O	9.277447	-1.923705	-1.424635
C	7.019625	-1.181978	-1.585167
H	7.210243	-0.674525	-2.523665
C	5.769051	-1.141716	-0.954718
S	4.291107	-0.371412	-1.517525

C	-0.188519	-0.898329	1.115299
H	-1.075164	-0.940382	1.721674
S	1.176147	-1.923356	1.372979
C	1.960124	-1.154025	-0.021471
C	3.264256	-1.639013	-0.329977
N	3.871613	-2.552006	0.436277
C	5.059783	-2.971461	-0.166694
C	5.869829	-4.029964	0.286049
H	5.546609	-4.594736	1.151714
C	7.039573	-4.341295	-0.369640
H	7.699842	-5.131213	-0.029646
C	7.491319	-3.603117	-1.507702
O	8.629711	-3.823167	-2.042960
C	6.613252	-2.579358	-2.016758
H	6.921494	-2.050546	-2.910500
C	5.426727	-2.295342	-1.337892
S	4.172161	-1.130586	-1.763370
